# Prenatally Detected Sub-amniotic Cysts: Are They Significant?

**DOI:** 10.7759/cureus.94989

**Published:** 2025-10-20

**Authors:** Nikolaos Antonakopoulos, Panagiota Tzela, Georgios Adonakis

**Affiliations:** 1 Department of Obstetrics and Gynecology, School of Health Sciences, University of Patras, Patras, GRC; 2 Department of Midwifery, School of Health and Care Sciences, University of West Attica, Athens, GRC

**Keywords:** fetal anemia, fetal growth restriction, placental cysts, subamniotic cysts, subamniotic hemorrhage, umbilical cord cysts

## Abstract

We report the case of a gravida 2, para 1 woman in whom multiple relatively large sub-amniotic cysts were identified during a routine second-trimester ultrasound. The lesions progressively enlarged but were not associated with abnormal Doppler findings or fetal growth restriction. A planned cesarean delivery at 37 weeks resulted in the birth of a healthy infant, and the cystic findings corresponded with the placental macroscopic appearance at delivery. This case highlights the sonographic features, differential diagnosis, and clinical implications of sub-amniotic cysts. Although the prognosis is generally favorable, careful serial monitoring is warranted in the presence of large or multiple lesions. Further studies are needed to establish standardized management protocols and evaluate long-term outcomes.

## Introduction

Sub-amniotic cysts are uncommon benign lesions of the umbilical cord or placenta, occurring in approximately 2-7% of pregnancies and typically identified incidentally during prenatal ultrasound or at delivery [1,2]. Their early incidence in the first trimester is extremely low (0.15%), according to recent data [3]. These fluid-filled lesions are located between the amnion and the fetal surface of the placenta. Although described several decades ago, their clinical significance remains poorly understood due to their rarity and frequent incidental detection. With the increasing use of high-resolution prenatal imaging, sub-amniotic cysts are being identified more often, primarily during the second and third trimesters (44% and 38%, respectively) [4]. However, their exact etiology is not yet fully elucidated. Despite their generally benign course, large or multiple cysts have been associated with adverse pregnancy outcomes, particularly fetal anemia and growth restriction [5,6].

Placental cystic lesions include several types (e.g., sub-amniotic, subchorionic, and septal cytotrophoblastic cysts), and their sonographic characteristics often overlap, making differentiation challenging. Sub-amniotic cysts, in particular, are defined by their position beneath the amnion on the fetal surface of the placenta. Studies suggest that when these cysts are large or located near critical structures, such as the umbilical cord insertion, they may cause a mass effect or alter placental hemodynamics, potentially contributing to growth restriction or fetal compromise.

Prenatal ultrasound diagnoses of sub-amniotic cysts or related sub-amniotic hematomas have been linked to elevated maternal serum markers and perinatal complications, including fetal growth restriction, mild fetal anemia, and preterm labor, in several case series [6,7].

Given the growing frequency of detection and the potential for adverse outcomes, further clarification is needed regarding their natural history, risk stratification, diagnostic features, and management. This manuscript presents a case of multiple relatively large sub-amniotic cysts without clinical complications and reviews the literature to better define prognostic factors and propose management strategies for these rare but potentially significant placental lesions.

## Case presentation

**Figure 1:**
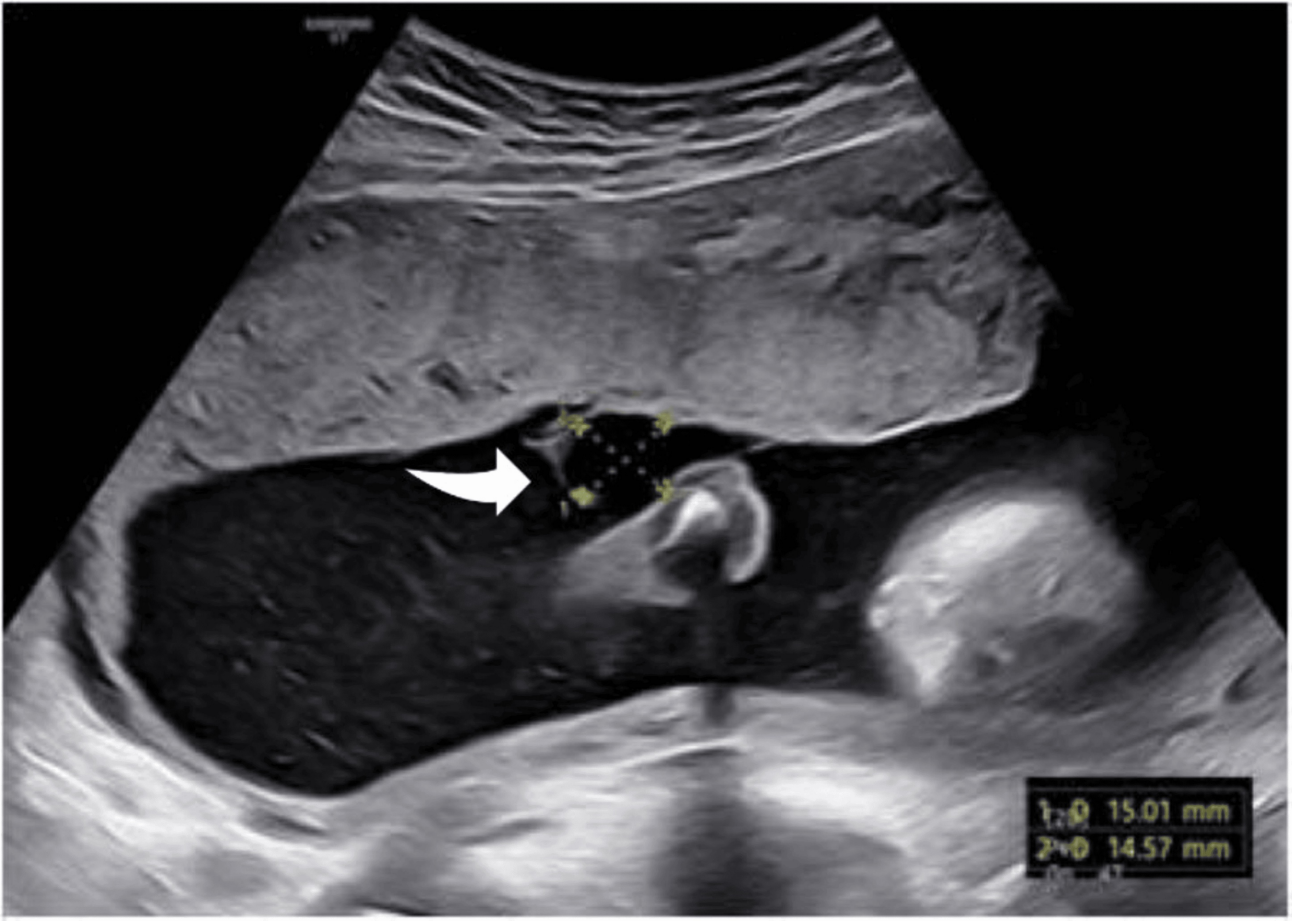
Appearance of the main sub-amniotic cyst (arrow) when first identified at 21 weeks of gestation

A gravida 2, para 1 woman with a history of a previous cesarean section underwent a routine second-trimester anatomy scan in our fetal medicine unit. The ultrasound revealed a small anechoic cystic structure measuring approximately 1.5 cm adjacent to the placental cord insertion (Figure 1). A diagnosis of a sub-amniotic cyst was made, and monthly follow-up ultrasounds were recommended to monitor the cyst, fetal growth, and Doppler findings.

**Figure 2:**
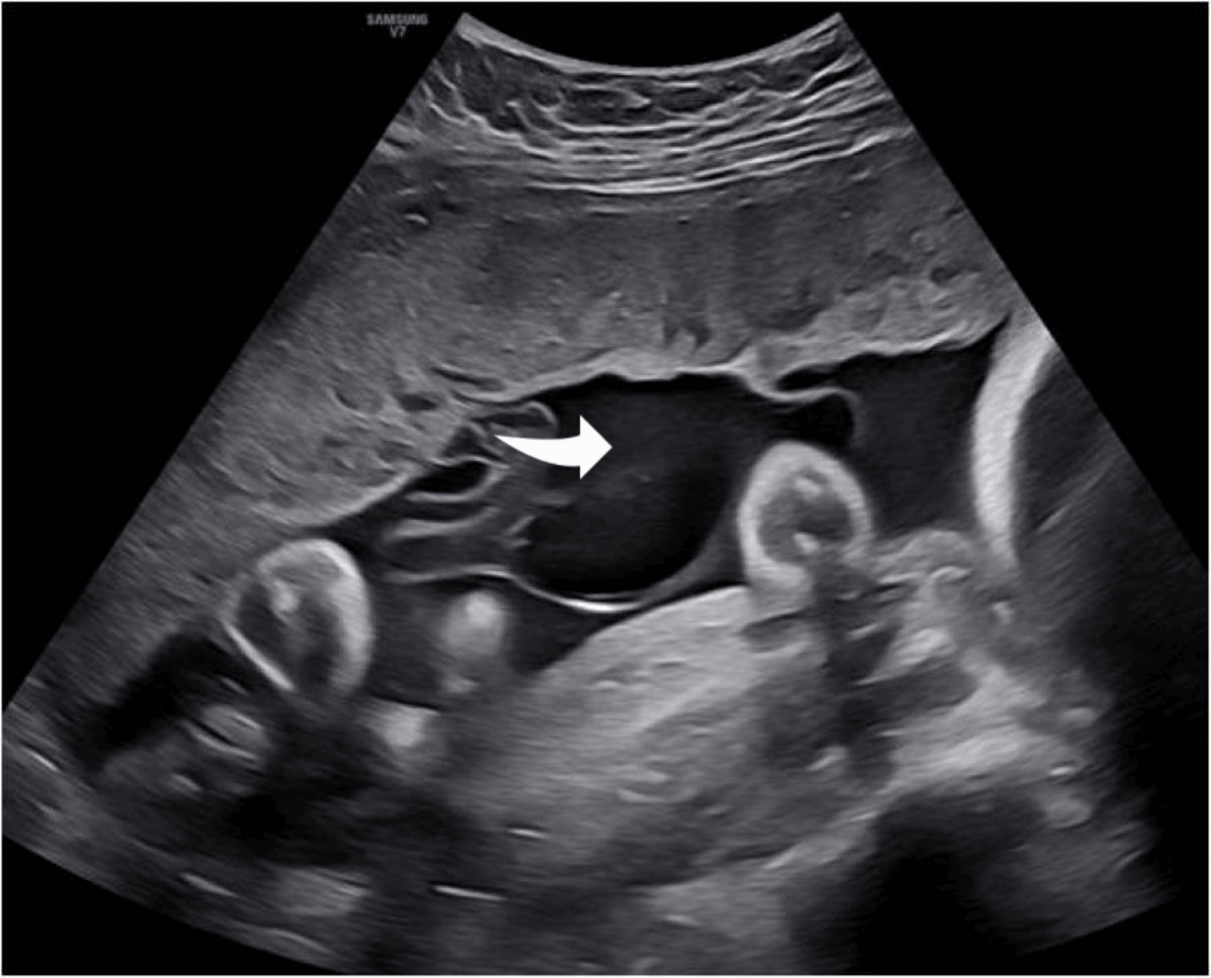
Appearance of the enlarged cyst (arrow) at 32 weeks

**Figure 3:**
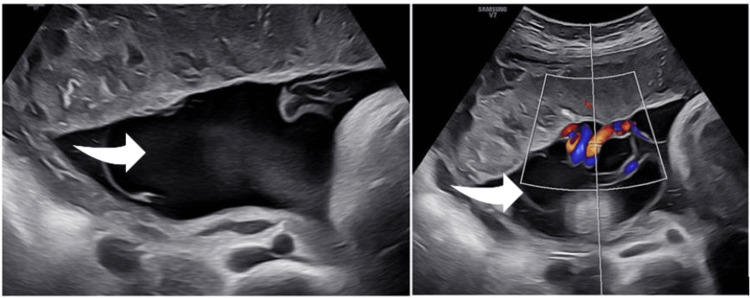
Appearance of the main sub-amniotic cyst (arrow) at 37 weeks, just prior to the planned cesarean section (left: longest axis view; right: adjacency to placental cord insertion)

**Figure 4:**
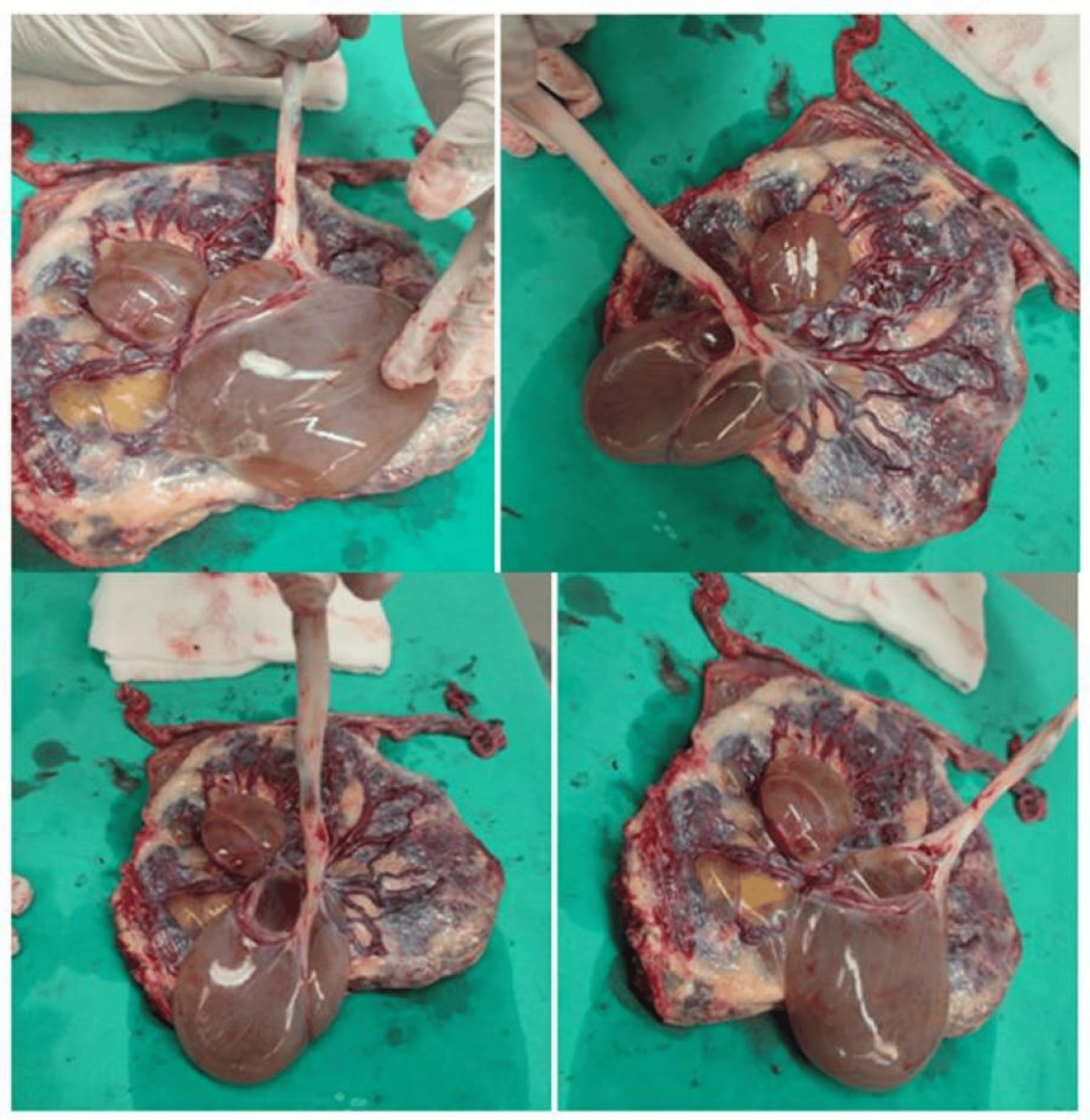
Placental sub-amniotic cysts from different viewing angles

The cyst continued to increase in size (Figure 2), but the fetal growth centile and Doppler findings remained normal throughout follow-up. Given the cyst’s size and the appearance of a smaller satellite cyst, a cesarean section was scheduled at 37 weeks of gestation after consultation and obtaining informed consent. The day before the operation, an ultrasound scan confirmed the size of the main cyst and its relationship to the placental cord insertion (Figure 3). The sonographic appearance of the cyst closely matched the macroscopic appearance of the placenta after delivery (Figure 4).

## Discussion

Sub-amniotic cysts are a rare prenatal sonographic finding. Their typical ultrasound appearance is that of anechoic, well-circumscribed lesions with no communication with the amniotic cavity [5]. Similar to our case, these cysts are most often located near the placental cord insertion, beneath the thin amniotic layer covering the chorionic plate, and they may protrude into the amniotic cavity. They can appear as single or multiple cysts, as in our patient. When color Doppler is applied, no blood flow is detected. Magnetic resonance imaging may be used in complex cases for better characterization.

Several hypotheses have been proposed regarding their origin, including degenerative changes in the amnion or Wharton’s jelly, obstructive phenomena due to abnormal umbilical vessel development or trauma, and localized fluid collection caused by rupture of sub-amniotic vessels [5]. Histologically, these cysts lack a true epithelial lining and are often classified as pseudocysts. Sub-amniotic cysts may contain fetal blood, as demonstrated by acid-elution testing and hemoglobin analysis, and in such cases, they are associated with fetal anemia and growth restriction [6]. This condition is defined as bleeding between the amnion and the chorionic plate, usually resulting from rupture of chorionic (fetal) vessels close to the umbilical cord insertion [7]. However, sub-amniotic hemorrhage more commonly occurs during delivery due to early or inappropriate cord traction [8].

Although sub-amniotic cysts are generally benign, complications such as umbilical vessel compression, fetal growth restriction, abnormal fetal heart rate patterns, and, in rare cases, stillbirth may occur [5]. In general, sub-amniotic hemorrhage has been associated with vaginal bleeding, feto-maternal hemorrhage, fetal growth restriction, fetal asphyxia, fetal anemia, and perinatal death, with fetal outcome largely dependent on the size of the hematoma [9]. Intrauterine growth restriction (IUGR) has been reported in approximately 10% of cases, and cysts larger than 4.5 cm or numbering more than three are more frequently linked to IUGR [10]. The peak systolic velocity of the middle cerebral artery serves as a predictor of fetal anemia; however, because of the concurrent risk of IUGR, this measurement should be interpreted with caution [11]. The cysts may rupture into the amniotic cavity, producing blood-stained, hyperechoic amniotic fluid. Once absorbed, sub-amniotic hemorrhages may appear as hyperechoic deposits on the fetal surface of the placenta [7].

The main differential diagnoses include placental cysts and large umbilical cord cysts near the placental insertion. True placental cysts (cytotrophoblastic cysts) may be located within the placental parenchyma or beneath the fetal plate; however, they are filled with gelatinous material rather than blood and are not typically associated with adverse perinatal outcomes. In contrast, umbilical cord cysts appear as anechoic structures, and when identified in the first trimester, particularly near the placental insertion, over 20% are associated with fetal chromosomal or structural abnormalities [12].

No standardized guidelines exist for the management of sub-amniotic cysts. Serial ultrasound scans are recommended to monitor cyst size and assess fetal well-being [5]. Delivery planning should be individualized based on fetal condition and cyst progression [5]. Prognosis is generally favorable, especially for isolated or small cysts; however, close surveillance is warranted in cases involving large or multiple cysts, abnormal fetal growth, or coexisting placental anomalies.

## Conclusions

This case highlights the importance of recognizing sub-amniotic cysts as a distinct entity among placental cystic lesions. Careful ultrasound characterization, accurate differentiation from other cyst types, and vigilant antenatal monitoring are essential to anticipate potential complications such as growth restriction, anemia, or preterm delivery. Multidisciplinary management is key to minimizing risks and promoting favorable maternal and fetal outcomes.

Given the limited number of reported cases, standardized management guidelines for sub-amniotic cysts are still lacking. Future research through larger case series and prospective studies is needed to clarify their natural history and establish evidence-based management protocols. In the meantime, individualized care, guided by precise diagnosis, ongoing monitoring, and shared decision-making, remains the most effective strategy.
